# A person‐centered and genetically informed approach toward characterizing multidomain resilience to neighborhood disadvantage in youth

**DOI:** 10.1111/jcpp.70068

**Published:** 2025-10-24

**Authors:** Jessica L. Bezek, Elizabeth A. Shewark, Gabriela L. Suarez, Kelly L. Klump, S. Alexandra Burt, Luke W. Hyde

**Affiliations:** ^1^ Department of Psychology University of Michigan Ann Arbor MI USA; ^2^ Department of Psychology University of Notre Dame Notre Dame IN USA; ^3^ Department of Psychology Michigan State University East Lansing MI USA; ^4^ Survey Research Center at the Institute for Social Research University of Michigan Ann Arbor MI USA

**Keywords:** Resilience, neighborhood, parenting, behavioral genetics, latent profile analysis

## Abstract

**Background:**

Examining resilience to adversity across multiple behavioral domains (e.g., psychological well‐being, social functioning) can better characterize positive development and inform novel prevention and intervention efforts. However, few studies have employed person‐centered methods to examine individual profiles of resilience across multiple domains in youth. Further, research exploring contextual predictors of resilience has rarely used genetically informed designs, which are critical for eliminating potential confounds.

**Methods:**

The current study employed latent profile analysis (LPA) to extract profiles of resilience across psychological, social, and academic domains in 708 adolescent twins exposed to neighborhood disadvantage, a pervasive form of early life adversity. Next, associations between profile membership and parenting, peer, and neighborhood social processes were examined. Lastly, co‐twin control analyses were conducted to explore whether associations between resilience profile membership and social processes were environmental versus genetic in origin.

**Results:**

Youth were grouped into three resilience profiles: (1) High Multidomain Resilience (63%), (2) Low Psychological Resilience, High Social Resilience (19%), and (3) Low Multidomain Resilience (18%). Profiles differed in experiences of parenting (i.e., parental involvement, conflict), peer characteristics (i.e., friend drug‐related behaviors, popularity), and neighborhood processes (i.e., social cohesion, informal social control, positive social norms). Follow‐up analyses within‐twin pairs revealed that the association between higher resilience and parenting (higher nurturance, lower conflict) was at least partially environmental in origin.

**Conclusions:**

Youth show distinct profiles of resilience across psychological, social, and academic domains, which are uniquely related to processes at the family, peer, and neighborhood level. Further, the association between resilience and parenting is in part environmentally mediated, suggesting a modifiable pathway to boost resilience in adolescents exposed to neighborhood disadvantage.

## Introduction

Neighborhood disadvantage is associated with greater risk for negative outcomes, including poorer physical health, mental illness, and worse academic performance (Hyde et al., 2020; Leventhal & Brooks‐Gunn, 2000). However, many youth exhibit resilience, defined as *positive* functioning in the face of adversity. Resilience research has high translational value, as identifying and boosting protective factors that foster resilience can support more adaptive outcomes for youth facing adversity.

Most research has defined resilience as the lack of psychopathology in the face of moderate to severe stress (Masten, 2001). However, recent theory and empirical work highlight that resilience encompasses both the lack of negative *and* the presence of positive functioning across multiple domains, including mental health, academic success, and social functioning (Bonanno, 2012; Infurna & Jayawickreme, 2019; Miller‐Graff, 2022). Interestingly, resilience in one domain may have only small to moderate correlations with resilience in another domain (Burt, Klump, Vazquez, Shewark, & Hyde, 2021), indicating that youth may show resilience in one area but not another.

Despite the emerging consensus that resilience is a multidimensional construct across the population, less is known about *within‐person* levels of resilience across behavioral domains in youth. That is, are there different patterns of resilience within individuals (e.g., showing resilience in one domain but not others)? Person‐centered analytic methods, such as latent profile analysis (LPA) (Bergman, Magnusson, & El Khouri, 2003; Ferguson, Moore, Hull, & Hull, 2020), can identify clusters of youth with distinct behavioral profiles and thus inform our understanding of how resilience varies across domains within a person. However, data clustering methods have primarily been used to analyze profiles of risk‐related outcomes (e.g., psychopathology) and thus typically identify only one ‘resilient’ profile (Brody et al., 2013; Cameranesi, Shooshtari, & Piotrowski, 2022).

Thus far, the few studies utilizing data‐driven clustering methods to identify profiles of resilience have revealed distinct combinations of cognitive, social, and behavioral resilience. These studies included children (3–5 years) in the welfare system (Yoon et al., 2022), children (5–11 years) exposed to low socioeconomic status and child protective services (Green et al., 2023; Herbers, Cutuli, Jacobs, Tabachnick, & Kichline, 2019; Yoon et al., 2021), and young adults emancipated from the foster care system (Yates & Grey, 2012). Notably, no studies have examined multidomain resilience profiles among adolescents, a surprising gap given the substantial biological changes characteristic of adolescence (Jaworska & MacQueen, 2015). Additionally, youth spend substantially more time outside of the home during adolescence, emphasizing the importance of studying neighborhood contexts (e.g., schools, peers). Given that no research has examined resilience profiles among adolescents exposed to neighborhood disadvantage, an exposure robustly linked to negative outcomes, our first aim was to investigate unique profiles of resilience in a relatively large sample of adolescents exposed to moderate to severe neighborhood disadvantage.

In addition to characterizing *what* resilience looks like at the person‐specific level, it is critical to identify factors associated with resilience, as these factors may reflect malleable experiences to target in future interventions. For adolescents, key social contexts include relationships with parents and peers (Laursen & Veenstra, 2021). Additionally, neighborhood social processes take on greater importance as teens spend more time outside of the home (Henry, Gorman‐Smith, Schoeny, & Tolan, 2014). Supportive parents, positive peers, and positive neighborhood social processes are well‐established promotive developmental experiences (Graham‐Bermann, Gruber, Howell, & Girz, 2009; Haskett, Nears, Ward, & McPherson, 2006; Yoon et al., 2022). Thus, our second aim sought to replicate and extend previous work by examining whether these environmental factors were related to person‐centered constellations of multidomain resilience.

Although existing work has identified meaningful associations between social factors and resilience, a major, often overlooked confound is gene–environment correlation. For example, warm parenting is associated with resilience, yet this association could also be due to shared genes between parents and children that underlie both positive parenting and resilience to adversity. Genetically informed designs are a powerful tool for ruling out genetic confounding and strengthening causal inference. As such, our third aim leveraged a twin design to test whether associations between resilience and social contexts observed in Aim 2 were due to genetic versus environmental influences (Plomin, 2008).

In sum, the current study utilized data‐driven clustering methods to identify profiles of resilience across social, academic, and psychological domains. Next, we examined associations among the identified profiles with processes at the level of parents (parental involvement and conflict), peers (friend delinquency, academics, popularity, and drug‐related behaviors), and the neighborhood (social cohesion, informal social control, and norms). Finally, using co‐twin control analyses, we examined whether experiences that differed among twins (i.e., parenting, peer relationships) were associated with resilience via genetic versus environmental pathways. We examined these questions in a unique cohort of twins sampled from birth records and exposed to above‐average levels of neighborhood disadvantage.

## Methods

### Participants

Participants were part of the Michigan Twins Neurogenetics Study (MTwiNS), recruited from the Twin Study of Behavioral and Emotional Development – Child (TBED‐C), a project within the Michigan State Twin Registry (Burt & Klump, 2013). The study identified twin families with children aged 6–10 years old via birth records living within 120 miles of East Lansing, MI, USA, including urban (e.g., Detroit, Flint, Lansing), suburban, and rural areas. TBED‐C included a population‐based arm (528 twin families) and an under‐resourced arm (502 twin families) from the same geographic region but recruited only from neighborhoods with above‐mean levels of family poverty for the state of Michigan at the time of recruitment (>10.5% of families in the neighborhood living below the poverty line) (Burt & Klump, 2013). Researchers followed up during adolescence with families from the under‐resourced arm and families from the population‐based arm that would have met criteria for the under‐resourced arm (i.e., living in disadvantaged neighborhoods). Participants included 708 twins from 354 families (54.7% male; 78.2% White, 12.7% Black, 1.1% Hispanic, and 7.5% ‘other’ ethnoracial identity). Youth were 7–19 years old, but almost all (94.2%) were 10–17 years old (*M*_age_ = 14.58, *SD* = 2.23), and most were 12–17 years old (84%). The mean neighborhood poverty level for families in the study was 20% (i.e., 20% of families' neighbors were living below the poverty line) and ranged up to 77%.

### Multidimensional resilience

Consistent with our previous work in this cohort (Bezek et al., 2024; Burt et al., 2021), we utilized data from multiple measures and informants to create a comprehensive measure of resilience across 10 unique indicators. Four participants were dropped due to missing all 10 indicators, resulting in a final sample of 704 youth.

#### Psychological resilience

Four indicators of psychological resilience were collected. Youth reported on *personal and relational resilience* and *life satisfaction* via the 17‐item Child and Youth Resilience Measure‐Revised (CYRM‐R; *α* = .90) (Jefferies, McGarrigle, & Ungar, 2019) and the 5‐item Satisfaction with Life Scale (SWLS; *α* = .89) (Diener, Emmons, Larsen, & Griffin, 1985), respectively. The other indicators included parent and youth reports of a *lack of psychopathology* using the eight psychopathology subscales from the Child Behavior Checklist (CBCL) and the Youth Self Report (YSR) (Achenbach & Rescorla, 2000). We recoded each scale as a dichotomous variable, indicating whether the child was resilient by being below (1) versus above (0) the ‘borderline’ cut‐point for clinical significance. The eight dichotomous variables were then summed to serve as our indicators of resilience to psychopathology, ranging from 0 to 8 (with eight indicating no psychopathology across all eight subscales).

#### Social resilience

Four indicators of social resilience were collected, including parent and youth reports on the *Social Activities* and *Social Competency* subscales of the CBCL and the YSR. These scales measure the child's involvement in clubs, activities, and organizations, as well as the number of friends, contact with friends, behavior alone, and behavior with others.

#### Academic resilience

Two indicators of academic resilience were collected, including teacher reports on the *Academic Performance* subscale of the Teacher Report Form (TRF; *α* = .94) and parent reports on the *School Competency* subscale of the CBCL. Each subscale's sum score was utilized to assess school performance across subjects, special education services received, repeated classes, and academic or other school‐related problems.

### Social context measures

#### Parenting relationship

The Parental Environment Questionnaire (PEQ) (Elkins, McGue, & Iacono, 1997) assessed parental involvement (i.e., parental nurturance; 12 items; *α* = .74) and conflict (12 items; *α* = .82) in each parent–child relationship. A sum score was calculated for the involvement (e.g., ‘I praise my child when he/she does something well’) and conflict subscales (e.g., ‘I often criticize my child’). Primary caregivers reported on each twin individually, and each twin reported on their parent.

#### Friend characteristics

Twins reported on the characteristics of their peer group via the 18‐item Friends Questionnaire (Walden, McGue, Burt, & Elkins, 2004). For each item, youth rated how many of their friends aligned with behaviors on four subscales: popularity (*α* = .78), drug‐related behaviors (*α* = .88), academics (*α* = .78), and delinquency (*α* = .82; see Appendix S1 for questionnaire details).

#### Neighborhood social processes

A randomly selected sample of participants' neighbors (*N* = 983) unrelated to participant families was recruited to serve as neighborhood informants on social processes (61% female, 34% male, 5% missing/prefer not to answer; 86.6% White; 6.7% Black; 4.6% other; 2.1% missing/prefer not to answer). Neighborhood informants completed the Neighborhood Matters Questionnaire, comprised of three subscales: social cohesion, informal social control, and norms (Henry et al., 2014). The 30‐item *Social Cohesion* scale assesses perceptions of neighborhood support, help, and trust. The 29‐item *Informal Social Control* scale assesses perceptions of the degree to which community residents will engage in activities maintaining social order. The 22‐item *Norms* scale assesses perceptions of neighborhood behavioral norms (e.g., child welfare, neighborhood safety).

Of the 704 twins in the sample, 570 twins had at least one neighborhood informant report on informal social control, and 572 twins had at least one neighborhood informant report on social cohesion and norms (*M* = 4.39 informant reports per neighborhood; *SD* = 1.64). The response rate was 51%, of which 62% agreed to participate (Appendix S1).

#### Sociodemographic variables

We examined profile differences in youth demographics (parent‐reported child age, race, and gender), as well as family and neighborhood‐level resources. Family income was measured as primary caregiver‐reported monthly household gross income and any additional outside sources of income (e.g., government assistance, child support). Youth also received an area deprivation index score, a metric characterizing neighborhoods' socioeconomic conditions using 17 census indicators such as income, education, and housing quality (Appendix S1, Figure S1; Kind et al., 2014).

### Latent profile analysis

We conducted a series of latent profile analyses with 10 resilience indicators (i.e., Psychological resilience: personal and relational resilience, satisfaction with life, parent‐ and youth‐reported lack of psychopathology; Social resilience: parent‐ and youth‐reported social activity engagement, parent‐ and youth‐reported social competency; Academic resilience: academic performance, school competency) in Mplus Version 8.11 (Muthén & Muthén, 2017). Four variance–covariance specification structures were tested (Johnson, 2021; Masyn, 2013), beginning with a one‐profile solution and including one additional profile per model until reaching stopping criteria (Table S2). Models were compared across three fit criteria: Bayesian Information Criterion (BIC), Sample‐Adjusted BIC, and Adjusted Lo‐Mendell‐Rubin Likelihood Ratio Test. Lastly, the theoretical distinctiveness of each additional profile was evaluated (Appendix S2).

#### BCH 3‐step follow‐up analyses

To assess mean differences between profiles on distal continuous outcomes, we conducted the Bolck, Croon, and Hagenaars (BCH) 3‐step method on the best‐fitting model (Asparouhov & Muthén, 2014). This method accounts for classification inaccuracy in profile membership when comparing profile means across outcome measures. To ensure that profile membership did not shift during the analysis, we included the means of auxiliary variables for each profile within BCH models.

We included all variables from each questionnaire within each model to control for the overlap of the other variables (e.g., the peers model included all four measures of peer characteristics). In total, we conducted four BCH outcome models: (1) Parent‐reported conflict and involvement, (2) Twin‐reported conflict and involvement, (3) Peer characteristics, and (4) Neighborhood social processes. Within every model, four covariates were included: parent‐report of twins' age, gender, race, and family income. We included race as a covariate to account for differences in exposure to structural racism and unequal exposures to poverty, stress, trauma, and opportunity for people of color living in the United States (Roberts & Rizzo, 2021).

### Twin difference and co‐twin control analyses

To assess whether associations between resilience profile membership, parenting, and peers were more genetic or environmental in origin, we conducted a series of co‐twin control analyses. Neighborhood characteristics were not compared because twins had the same neighborhood scores within each family.

First, we computed twin difference scores for resilience, parent, and peer variables. Twin profile membership was treated as an ordinal variable, such that *Low Multidomain Resilience* was coded as one, *Low Psychological, High Social Resilience* was coded as two, and *High Multidomain Resilience* was coded as three. Within each twin pair, twin profile membership scores were subtracted to create a resilience difference score ranging from −2 (twin 2 more resilient than twin 1) to 2 (twin 1 more resilient than twin 2). We then calculated twin difference correlations separately by zygosity, thus representing the relationship between resilience profile and each environmental exposure within monozygotic and dizygotic twin pairs.

**Figure 1:**
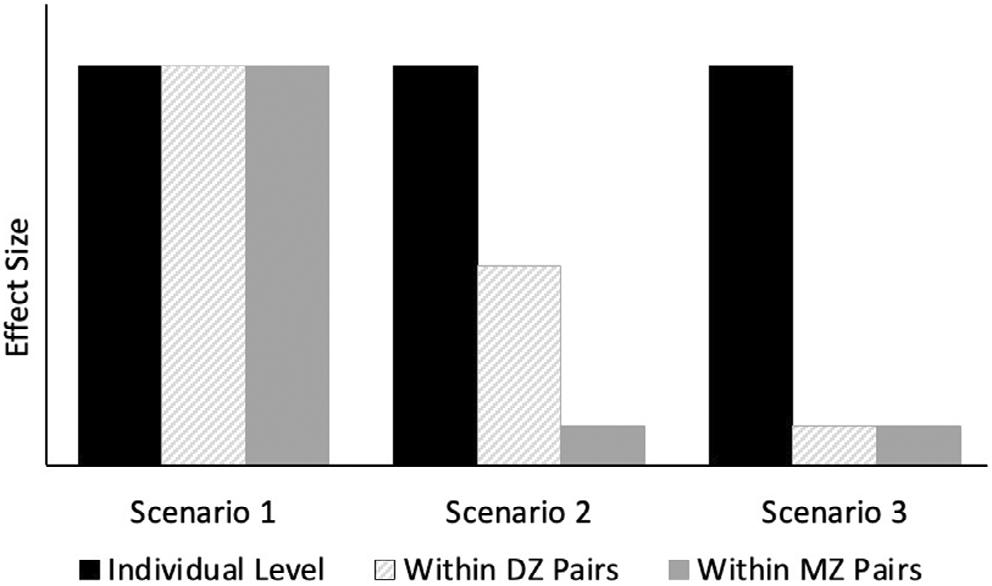
Hypothetical co‐twin control scenarios. Hypothetical results for the association between resilience and social exposures across twin pairs. *Scenario 1*: The association between resilience and social exposure is solely environmental in origin. In this scenario, the effect size of the relationship between resilience and the social exposure is equivalent at the individual level (i.e., between all individuals in the sample), within dizygotic pairs who are discordant on the social exposure, and within monozygotic pairs who are discordant on the social exposure. *Scenario 2*: The association between resilience and social exposure is attributable to genetic confounds. In this scenario, resilience is associated with a given social exposure only at the individual level or within dizygotic twin pairs, but not within monozygotic twins. *Scenario 3*: The association is both genetic and shared environmental in origin. In this scenario, resilience is only associated with the social exposure at the individual level but not within‐twin pairs. DZ = dizygotic; MZ = monozygotic. Figure adapted with permission from Burt et al. (2021). See McGue et al. (2010) for more information on co‐twin control analysis and interpretation

Next, we conducted co‐twin control analyses in SPSS version 29.0.2 (IBM Corp., 2023). In this analysis (which is similar to the idealized counterfactual model of causation; McGue, Osler, & Christensen, 2010), we utilize the more exposed co‐twin's score to estimate the less exposed co‐twin's score had they experienced higher levels of the environmental exposure. For example, is the twin with higher exposure to a protective factor than their co‐twin also higher on resilience? For each parenting and peer variable, we conducted three multilevel models relating resilience to the environmental exposure: (1) Within the entire sample, (2) Within only monozygotic twin pairs, (3) Within only dizygotic twin pairs. We then compared results to three potential outcomes reflecting different combinations of genetic and environmental effects (Figure 1; Appendix S2).

## Results

### Resilience latent profiles

#### Model fit characteristics

**Figure 2:**
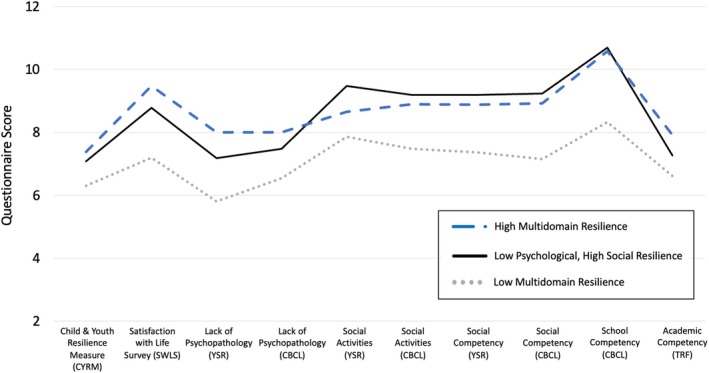
Youth exhibit three resilience latent profiles across psychological, social, and academic resilience domains. Evaluation of model fit statistics across one‐, two‐, three‐, and four‐profile solutions revealed that the three‐profile model with profile‐specific variance and no covariances fit the data best. The three resilience profiles were labeled as (1) Low Multidomain Resilience, (2) Low Psychological, High Social Resilience, and (3) High Multidomain Resilience. The Low Multidomain Resilience Profile reported poorer functioning on all 10 indicators of resilience across psychological, social, and academic domains. The Low Psychological, High Social Resilience exhibited lower levels of psychological resilience as well as lower academic competency compared to youth in the High Multidomain Resilience group. However, these youth reported the highest activity engagement and equally high social resilience to peers in profile three. The High Multidomain Resilience group showed the highest levels of psychological resilience compared to both other profiles, as well as the highest academic performance as rated by teachers. Child‐reported forms include the CYRM, SWLS, and YSR. Parent‐reported forms include the CBCL. Teacher‐reported forms include the TRF. CBCL: Child Behavior Checklist; TRF: Teacher Report Form; YSR: Youth Self‐report Form. The *Y* axis represents individuals' scores on each questionnaire. In order to create a similar range for all measures and more readily compare questionnaire scores across profiles, we rescaled four variables. Specifically, we divided the youth's total scores for the Child & Youth Resilience Measure by 10, divided youth's scores on the Satisfaction with Life Scale by three, multiplied parents' scores on the School Competency measure by two, and multiplied teachers’ scores on the Academic Competency measure by two

Four variance–covariance matrix specifications were evaluated to identify the best‐fitting model: (1) Equal variances, No covariances; (2) Profile‐specific variances, No covariances; (3) Equal variances, Equal covariances; and (4) Profile‐specific variances, Profile‐specific covariances (Table S1). According to model fit criteria, solutions from the profile‐specific variances, no covariances models fit the data best (Table S2). We compared one‐, two‐, three‐, and four‐profile solutions with profile‐specific variances and no covariances (Table 1). According to model fit criteria and theoretical considerations, the three‐profile solution fit the data best (Figure 2, Appendix S2).

**Table 1:** Latent profile analysis model fit statistics

Number of profiles	Number of parameters	Log likelihood	Bayesian Information Criterion (BIC)	Sample‐adjusted BIC	Entropy	Adjusted Lo Mendell Rubin Likelihood Ratio Test (LRT)
1‐profile	20	−9,439.981	19,011.097	18,947.592	–	–
2‐profile	39	−6,354.909	12,965.532	12,841.699	0.986	0.0000
**3‐profile**	**60**	**−6**,**179.829**	**12**,**753.065**	**12**,**562.552**	**0.924**	**0.0003**
4‐profile	81	−6,108.593	12,748.285	12,491.093	0.907	0.6644

*N* = 704. All models were run with profile‐specific variances and no covariances. Values are bolded indicates the model that was selected.

An analysis of variance was conducted in R (version 4.1.1) to compare each resilience profile, revealing that the profiles differed significantly across all 10 resilience indicators (Table 2). Profile one, ‘Low Multidomain Resilience,’ included youth with significantly lower resilience across all 10 indicators compared to profiles two and three. Profile two, ‘Low Psychological Resilience, High Social Resilience,’ exhibited lower scores on all psychological resilience measures and lower teacher‐reported competency compared to profile three. However, youth in profile two showed equivalent levels of social resilience compared to youth in profile three, as well as the *highest* youth‐reported activity engagement. Lastly, profile three, ‘High Multidomain Resilience,’ exhibited the highest levels of resilience across all four psychological resilience indicators and on teacher‐reported academic competency.

**Table 2:** Three‐profile solution analysis of variance (ANOVA) comparison of resilience indicators

	Profile 1: Low Multidomain Resilience (*n* = 127)		Profile 2: Low Psychological, High Social Resilience (*n* = 135)		Profile 3: High Multidomain Resilience (*n* = 442)		*df*	*F*	*η* ^2^	Tukey post‐hoc differences
	M	*SD*	M	*SD*	M	*SD*				
Child & Youth Resilience Measure	6.301	1.090	7.082	0.815	7.382	0.825	691	152.4***	.18	1 ≠ 2 ≠ 3
Satisfaction with Life Survey	7.190	2.290	8.778	1.553	9.474	1.611	446	121.8***	.21	1 ≠ 2 ≠ 3
Lack of Psychopathology (YSR)	5.813	2.003	7.184	0.649	8.000	0.032	689	592.0***	.46	1 ≠ 2 ≠ 3
Lack of Psychopathology (CBCL)	6.549	1.714	7.481	0.559	8.000	0.032	693	397.7***	.34	1 ≠ 2 ≠ 3
Social Activities (YSR)	7.857	2.494	9.476	2.122	8.660	2.449	455	4.191*	.01	1 ≠ 2 ≠ 3
Social Activities (CBCL)	7.482	2.862	9.196	2.316	8.897	2.195	454	20.29***	.04	1 ≠ 2, 3
Social Competency (YSR)	7.359	2.504	9.194	2.338	8.880	2.465	455	23.85***	.05	1 ≠ 2, 3
Social Competency (CBCL)	7.159	2.898	9.228	2.315	8.925	2.405	453	30.83***	.06	1 ≠ 2, 3
School Competency (CBCL)	8.324	2.649	10.697	1.267	10.583	1.649	454	92.11***	.17	1 ≠ 2, 3
Academic Performance (TRF)	6.613	1.951	7.261	1.915	7.911	1.899	336	23.64***	.07	1, 2 ≠ 3

CBCL, Child Behavior Checklist; *M*, mean, *SD*, standard deviation; TRF, Teacher Report Form; YSR, Youth Self‐report Form.

Statistically significant, *p < .05, ***p < .001.

### Demographic characteristics of resilience profiles

Analyses using the BCH 3‐Step method revealed that youth across the three profiles did not differ by gender or racial/ethnic group (Table 3). However, youth in the Low Multidomain Resilience profile were significantly older and had lower parent‐reported family income than the other two profiles. Lastly, youth in the High Multidomain Resilience profile had significantly lower neighborhood area deprivation index scores than youth in the Low Multidomain Resilience profile (i.e., those with high multidomain resilience lived in less disadvantaged neighborhoods). Given the difference between groups in neighborhood disadvantage, we conducted sensitivity analyses in a subsample of youth with more serious exposure to neighborhood disadvantage (i.e., area deprivation index scores of 30 or greater; Suarez et al., 2025). These analyses (*N* = 636) showed nearly identical profiles with almost identical group assignments. Moreover, the profiles did not differ by neighborhood disadvantage, demonstrating that the same profiles emerge in a sample with more homogeneous exposure to greater neighborhood disadvantage (Appendix S4, Figure S2, Tables S3–S6).

**Table 3:** Bolck, Croon, and Hagenaars (BCH) 3‐step analysis for demographic characteristics and parenting, peer, and neighborhood social processes

	Low Multidomain Resilience *(Profile 1)* vs. Low psych resilience, high social resilience *(Profile 2) estimate*	Low Multidomain Resilience *(Profile 1)* vs. High Multidomain Resilience *(Profile 3)* estimate	Low psych resilience, high social resilience *(Profile 2)* vs. High Multidomain Resilience *(Profile 3)* estimate
Demographic characteristics			
Age	0.831*	0.675**	−0.156
Gender	0.062	0.068	0.006
Race	0.016	0.021	0.005
Family Income	−1.230*	−1.165**	0.065
National Area Deprivation Index Score	4.482	5.793*	1.312
Environmental variables			
Neighborhood Social Cohesion	25.473^**†**^	3.404	−22.070*
Neighborhood Norms	15.273*	1.096	−14.176*
Neighborhood Informal Social Control	8.349*	3.998	−4.351
Parental Involvement (youth‐report)	−27.797**	−11.904^**†**^	15.893*
Parental Involvement (parent‐report)	−3.463	−1.008	2.455
Parental Conflict (youth‐report)	15.236	13.207	−2.029
Parental Conflict (parent‐report)	−7.419	4.379	11.797*
Friend Popularity	−3.661	−3.783*	−0.122
Friend Academics	−4.797	−2.399	2.398
Friend Delinquency	1.935	1.574	−0.361
Friend Drug‐taking Behaviors	−4.127^**†**^	−4.048**	0.079

BCH 3‐step models comparing mean levels of demographic characteristics and environmental variables across the three latent profiles. For each column with the format A profile versus B profile, negative values indicate that the A profile scored lower than the B profile. Positive values indicate that the A profile scored higher than the B profile. Note that the Low Multidomain Resilience profile shows lower scores on the friend popularity and drug‐taking behaviors subscales because the scale is scored such that lower scores indicate *more* of the youth's friends demonstrate the behavior.

*p < .05, **p < .01, ^**†**^.05 < *p* < .07.

### Environmental correlates of resilience profiles

#### Parenting relationship

**Figure 3:**
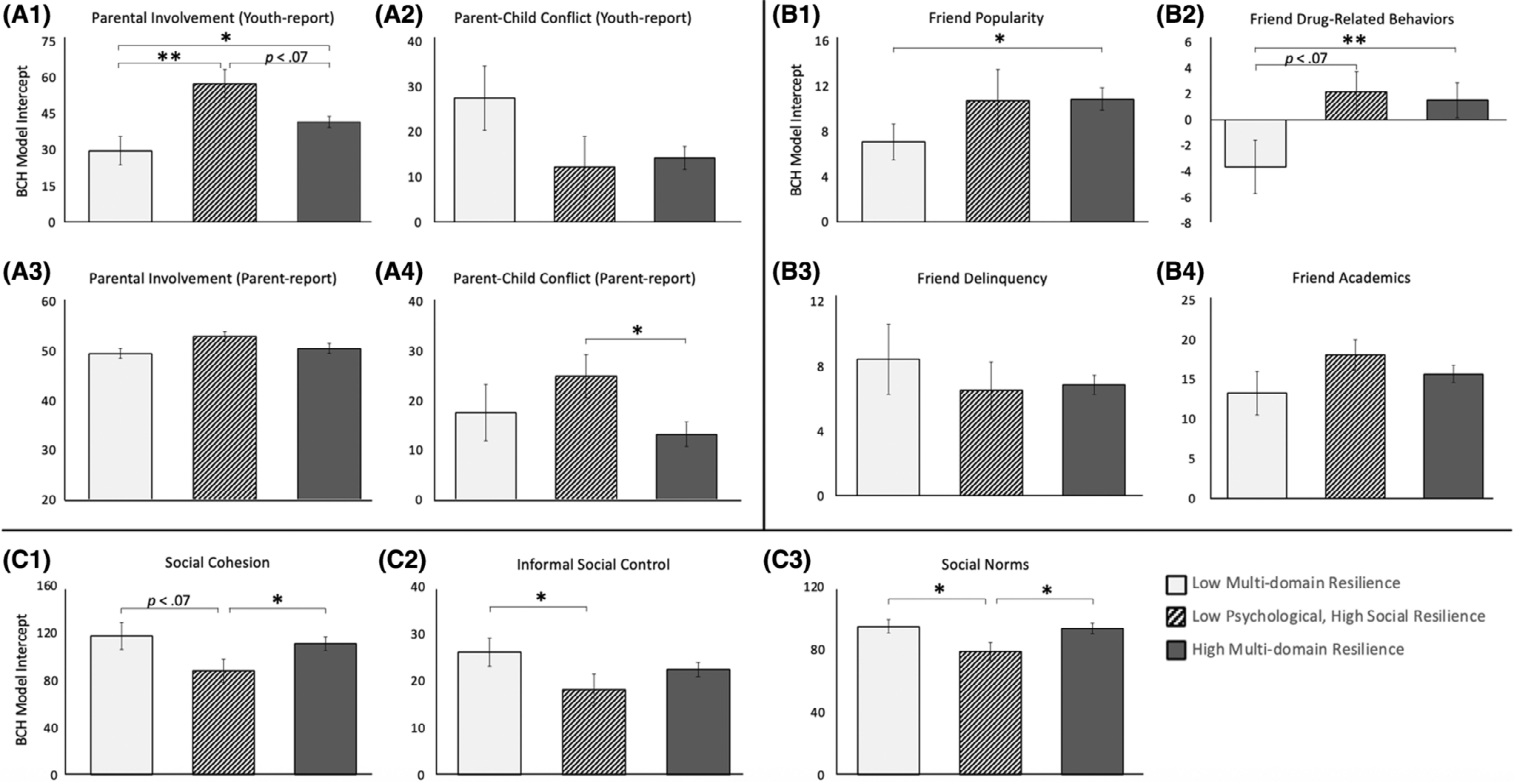
Resilience latent profiles differ in parenting, peer, and neighborhood social processes. Bolck, Croon & Hagenaars (BCH) 3‐step analyses showed statistically significant differences between resilience profiles on multiple measures of parenting, peer characteristics, and neighborhood social processes. Analyses included parent‐reported participant age, sex, race/ethnicity, and family income as covariates. Analyses account for classification inaccuracy by accounting for class membership probability when comparing profiles on social exposures. Graphs A1–A4 depict comparisons of resilience profiles across parental involvement and conflict as reported by parents and youth. Graphs B1–B4 depict comparisons of resilience profiles across four measures of friend characteristics. Graphs C1–C3 depict comparisons of resilience profiles across three neighborhood social processes. Youth in the High Multidomain Resilience profile showed significantly lower friend popularity (B1) and friend drug‐related behaviors (B2) than youth in the Low Multidomain Resilience Profile. Due to reverse coding on the friends questionnaire, youth reporting lower scores have *more* friends engaging in those behaviors. Youth in the High Multidomain Resilience profile showed significantly greater neighborhood social cohesion (C1) and norms (C3) than youth in the Low Psychological, High Social Resilience Profile. Youth in the Low Psychological, High Social Resilience Profile showed marginally greater twin‐reported parental involvement (A1) and significantly greater parent‐reported parental conflict (A4) compared to youth in the High Multidomain Resilience Profile. Youth in this profile also showed significantly greater twin‐reported parental involvement (A1) and lower informal social control (C2) and norms (C3) compared to the Low Multidomain Resilience profile. No other significant differences were observed between profiles. On the *y*‐axis, all graphs report latent profile intercepts on each social exposure variable. *p < .05, **p < .01

Parents of youth in the High Multidomain Resilience profile reported significantly less parent–child conflict than parents of youth in the Low Psychological, High Social Resilience profile (*p* < .05; Figure 3A). Additionally, there was a trend toward greater twin‐reported parental nurturance in the High Multidomain Resilience group compared to the Low Multidomain Resilience profile (*p* = .089). Interestingly, youth in the Low Psychological, High Social Resilience profile reported the greatest parental nurturance compared to youth in the other two profiles (*p* < .05; Figure 3A).

#### Friend characteristics

Youth in the High Multidomain Resilience profile reported having fewer friends engaging in drug‐related behaviors and a lower rating of friend popularity than those in the Low Multidomain Resilience profile (Figure 3B). Similarly, youth in the Low Psychological, High Social Resilience profile reported having fewer friends engaging in drug‐related behaviors than those in the Low Multidomain Resilience profile. No differences were observed between the High Multidomain Resilience profile and the Low Psychological, High Social Resilience profile on any friend characteristics. Profiles did not differ in friend academics or delinquency.

#### Neighborhood social processes

Youth in the High Multidomain Resilience profile resided in neighborhoods with higher social cohesion and stronger social norms regarding community and child safety than their peers in the Low Psychological, High Social Resilience profile (*p* < .05; Figure 3C). Similarly, youth in the Low Multidomain Resilience profile resided in neighborhoods with greater norms (*p* < .05) and greater informal social control (*p* < .05) than youth in the Low Psychological, High Social Resilience profile (Figure 3C). Unexpectedly, youth in the High Multidomain Resilience group did *not* differ from youth in the Low Multidomain Resilience profile on any neighborhood processes. Sensitivity analyses in the subsample of youth with ADI scores of 30 or greater also yielded a very similar pattern of findings across parent, peer, and neighborhood experiences (Table S6).

### Genetic vs. environmental contributions of social processes to resilience profile membership

Twin difference score correlations are reported separately by zygosity in Table 4 for each of the parenting and peer variables that significantly differed phenotypically by profile in BCH analyses. Within monozygotic and dizygotic twin pairs, the twin with greater resilience was the twin with lower parent‐reported parent–child conflict and higher twin‐reported parental nurturance. These findings offer preliminary evidence for a meaningful environmental effect. Otherwise stated, the monozygotic twin exposed to lower levels of harsh parenting exhibited greater resilience than their genetically identical co‐twin. Given identical DNA in monozygotic twins, this association must be environmental in origin. Despite observing phenotypic correlations between resilience and friend characteristics across the entire sample, we observed no significant twin difference correlations between resilience and friend popularity or drug‐related behaviors within monozygotic or dizygotic pairs.

**Table 4:** Twin difference correlations between resilience, parenting, and peer characteristics within‐twin pairs

	Monozygotic twins		Dizygotic twins	
	*r*	*p*‐Value	*r*	*p*‐Value
Parent–Child Conflict (parent‐report)	−0.196	.032	−0.149	.039
Parental Involvement (youth‐report)	0.246	.007	0.193	.008
Friend Drug‐related Behaviors	0.022	.813	0.011	.876
Friend Popularity	0.026	.773	0.107	.138

Values represent within‐twin pair correlations for the four social exposures that differed across profiles as identified using the BCH 3‐step method. For parent–child conflict, both monozygotic and dizygotic twin pairs show a significant negative correlation, revealing that lower parent–child conflict is associated with higher resilience. For parental nurturance, both monozygotic and dizygotic twin pairs show a significant positive correlation, revealing that higher parental nurturance is associated with higher resilience.

**Figure 4:**
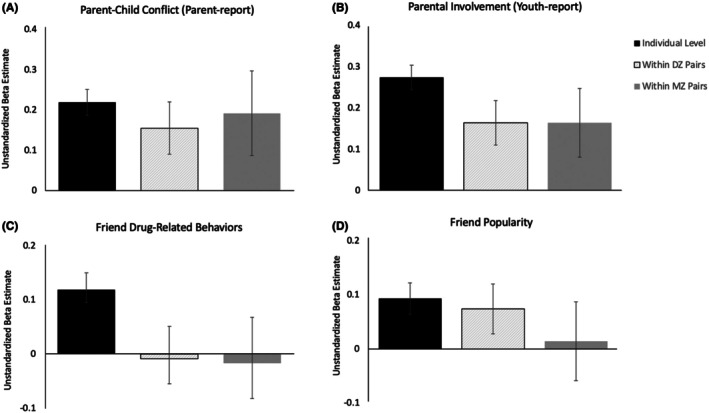
Co‐Twin control analyses identify environmentally mediated effects of parenting and mixed genetic and environmental effects of peers on resilience. (A) Unstandardized beta estimates for the association between resilience and parent–child conflict at the individual level, within all dizygotic twin pairs, and within all monozygotic twin pairs. Results are consistent with scenario one, in which the association between parent–child conflict and resilience is solely environmental in origin. *Z*‐score calculations exploring the difference in unstandardized betas for each group revealed no significant group differences (Individual level vs. DZ: *z* = −0.88, Individual level vs. MZ: *z* = −0.25, MZ vs. DZ: *z* = 0.30). The sign of the beta estimates are flipped for visualization purposes. Original estimates are negative given that parent‐child conflict is a negative exposure and predicts less resilience. (B) Unstandardized beta estimates for the association between resilience and parental involvement (i.e., parental nurturance) at the individual level, within all dizygotic twin pairs, and within all monozygotic twin pairs. Results are consistent with scenario one, in which the association between parental involvement and resilience is solely environmental in origin. *Z*‐score calculations exploring the difference in unstandardized betas for each group revealed no significant group differences at an alpha level of .05 (Individual level vs. DZ: *z* = 1.78, Individual level vs. MZ: *z* = 1.25, MZ vs. DZ: *z* = 0.00). (C) Unstandardized beta estimates for the association between resilience and friend drug‐related behaviors at the individual level, within all dizygotic twin pairs, and within all monozygotic twin pairs. Results are consistent with scenario three, in which the association between friend drug‐related behaviors and resilience is due to genetic and shared environmental confounds. *Z*‐score calculations exploring the difference in unstandardized betas for each group revealed that the individual‐level group does not significantly differ from the within‐dizygotic or within‐monozygotic groups at the .05 threshold, though this is likely due to the amount of standard error for within‐twin models (Individual level vs. DZ: *z* = −1.89, Individual level vs. MZ: *z* = −1.50, MZ vs. DZ: *z* = −0.25). The sign of beta estimates are flipped for visualization purposes. (D) Unstandardized beta estimates for the association between resilience and friend popularity at the individual level, within all dizygotic twin pairs, and within all monozygotic twin pairs. Results are consistent with scenario two, in which the association between friend popularity and resilience is due to genetic confounds. *Z*‐score calculations exploring the difference in unstandardized betas for each group are as follows: Individual level vs. DZ: *z* = 0.35, Individual level vs. MZ: *z* = 1.01, MZ vs. DZ: *z* = 0.70

Using a multilevel‐model regression framework, co‐twin control analyses revealed environmental mediation of the relationship between resilience and parenting (Figure 4). For parent‐reported parent–child conflict, the effect size of the association between parenting and resilience was not statistically different between models assessing individual differences across all youth in the sample, within MZ twins specifically, or within DZ twins (Figure 4A; Table S8). This pattern is most consistent with hypothetical scenario one (Figure 1), in which the association between resilience and parenting is solely environmental in origin. For twin‐reported parental nurturance, our results are also consistent with scenario one, in which the association between resilience and parental nurturance is environmentally mediated (Figure 4B; Table S8).

Peer characteristics revealed different patterns of environmental and genetic origin. For friend drug‐related behaviors, our results are most consistent with scenario three (see Figure 1) in which resilience was associated with friend drug‐related behaviors at the individual level, but not within MZ or DZ pairs (Figure 4C; Table S8). Thus, the causal mechanisms for this association are thought to be both genetic and shared environmental in origin. Lastly, associations between resilience and friend popularity were significant at the individual level and within dizygotic pairs but *not* within monozygotic pairs (Figure 4D; Table S8). These results are most consistent with scenario two (see Figure 1), in which the association between resilience and friend popularity likely reflects genetic confounds.

## Discussion

Among youth exposed to above‐average neighborhood disadvantage, we utilized LPA to extract profiles of resilience and explored profile differences in parent, peer, and neighborhood experiences. Additionally, we leveraged a twin study design to investigate whether associations between twins' resilience profiles and social experiences were more genetic or environmental in origin. Youth fit into three resilience profiles with consistently high, consistently low, or mixed resilience across psychological, social, and academic domains. Each resilience profile was associated with a unique combination of parenting, peer, and neighborhood social experiences, revealing the importance of characterizing multisystemic influences. Youth showing resilience in social but not psychological domains were most distinguished by neighborhood processes, highlighting lower levels of positive neighborhood social processes as an important risk factor for this group. Lastly, co‐twin control analyses revealed that the association between high multidomain resilience and parenting was at least in part environmental in origin, indicating that increasing parental nurturance and reducing parent–child conflict may be a particularly modifiable pathway for boosting adolescent resilience in the face of neighborhood disadvantage.

Most youth (63%) fell into a profile exhibiting high resilience across all domains, which aligns with evidence that many youth exposed to adversity exhibit resilience (Masten, 2001). However, 18% of the sample also exhibited low multidomain resilience, supporting evidence for the deleterious impact of neighborhood disadvantage on psychological, social, and academic outcomes (Hyde et al., 2020). Additionally, 19% of youth evidenced high social resilience (i.e., high activity engagement and social competency) but low psychological resilience (e.g., greater psychopathology, lower life satisfaction). This profile aligns with previous resilience clustering studies identifying a group with poorer emotional well‐being but higher resilience in another area, such as social abilities (Yoon et al., 2022) or occupational competence (Yates & Grey, 2012).

Consistent with hypotheses, resilience profiles differed in levels of parental nurturance and parent–child conflict. Youth in the High Multidomain Resilience profile had lower parent‐reported levels of parent–child conflict than youth in the Low Psychological, High Social Resilience profile – a finding consistent with evidence for the negative influence of harsh parenting on risk for psychopathology (Bender et al., 2007). However, youth in the Low Psychological, High Social Resilience profile reported greater parental nurturance compared to youth exhibiting resilience in all domains. Our results highlight that youth's perception of increased parental nurturance may help them do well in some areas (e.g., socially, academically), but potentially less so psychologically. Alternatively, youth who are functioning well in many domains but experiencing psychopathology may elicit greater warmth from their parent than youth who are functioning well in all or few areas. Importantly, results were specific to the reporter: only youth reports of positive parenting and parent reports of negative parenting differed across profiles. These results align with robust evidence for discordance in parent and adolescent reports on parenting (Hou et al., 2020), while raising the hypothesis that different facets of the parent–child relationship may be uniquely salient for specific outcomes (e.g., psychological well‐being). Despite reporter differences, co‐twin analyses revealed that the associations between resilience and parent–child conflict and involvement were not due to genetic confounding. Greater resilience was associated with lower levels of parent–child conflict and higher levels of parental nurturance over and above genetic and shared‐environment confounds, suggesting an environmental pathway. Thus, strengthening the parent–child relationship—even during adolescence, when youth are more frequently out of the home—may be a particularly powerful target for resilience‐oriented interventions.

At the level of peers, having friends with lower popularity who engage in fewer drug‐related behaviors appeared to distinguish youth with higher resilience. Previous work suggests that more popular youth are also less prosocial (Cillessen & Mayeux, 2004; Dumas, Davis, & Ellis, 2019). Thus, engaging with less popular, and thus potentially more supportive, friends may help promote resilience across multiple behavioral domains. Additionally, our results complement existing work that has identified peer substance use as a risk factor for negative adolescent outcomes (Duan, Chou, Andreeva, & Pentz, 2009). However, it's also possible that youth in more resilient profiles are choosing friends who aren't using substances (i.e., social selection) (Young, 2011). Using co‐twin control analyses, we show that the association between resilience, friend drug‐taking, and popularity is likely a combination of genetic and shared environment confounds. Thus, youth with genes supporting resilience may be selecting more supportive and adaptive friends, who in turn serve as environmentally mediated protective factors.

At the broadest level, neighborhood social processes most distinguished youth in the Low Psychological, High Social Resilience profile. Youth in this profile resided in neighborhoods with the lowest levels of social cohesion and positive social norms—two protective processes that mediate associations between neighborhood disadvantage and behavioral outcomes (Aneshensel & Sucoff, 1996; Sampson, Morenoff, & Gannon‐Rowley, 2002). Given that youth in the Low Psychological, High Social Resilience profile reported the highest activity engagement, it could be that elevated social engagement in a context with fewer neighborhood protective processes may be especially deleterious for adolescent mental health. Surprisingly, youth in the High Multidomain and Low Multidomain Resilience profiles did not differ in neighborhood processes, suggesting that neighborhood features cannot solely distinguish across resilience outcomes, at least in this sample where neighborhood disadvantage was elevated for all youth.

Though our study includes several strengths, such as a person‐centered approach, genetically informed design, and a well‐sampled cohort exposed to neighborhood disadvantage, some limitations exist. First, the study is cross‐sectional, precluding us from identifying the direction of associations between protective factors, such as lower parent–child conflict, and higher resilience. Longitudinal designs are necessary to test the direction of causality and provide the strongest evaluation of co‐twin causal inference. Further, the categorical nature of co‐twin control analyses resulted in smaller sample sizes for certain subgroups (e.g., discordant monozygotic twins), emphasizing the importance of large sample sizes for future genetically informed studies. Second, we focused on resilience to a specific form of adversity (i.e., neighborhood disadvantage) in a sample of youth representative of their home state, yet this sample may not generalize fully to other forms of adversity or regions. Third, all youth in the sample were exposed to neighborhood disadvantage broadly, yet research often explores resilience to specific adversity exposures. To address this issue, we re‐ran our LPA with youth in the sample who showed exposure to a high accumulation of specific adversities (e.g., community violence, abuse; Appendix S3; Table S7), as well as youth with more severe exposure to neighborhood disadvantage (Appendix S4; Tables S3–S5), and found similar profiles. Lastly, though our person‐centered approach offers a novel way to examine differing resilience profiles, resilience is a dynamic and dimensional process, which cannot be fully captured in latent groupings. Future combinations of variable‐ and person‐centered research measuring resilience dynamically over time will further advance our understanding of resilience.

## Conclusion

The current study offers novel insight into person‐centered profiles of psychological, social, and academic resilience within an adolescent cohort. We reveal associations between resilience and social processes across three levels of the environment to conclude that increasing parental involvement and reducing parent–child conflict may be particularly important environmentally mediated mechanisms for future interventions aimed at enhancing resilience in youth residing in disadvantaged neighborhoods.

## Ethical considerations

Participants' guardians provided informed consent, participants provided assent, and study methods were approved by the University of Michigan Institutional Review Board on 12/04/2019 (HUM00163965).

**Key points**

**What's known**

- Many youth exhibit resilience in the face of adversity. Updated theoretical models suggest that resilience can manifest across multiple areas of functioning.

**What's new**

- We utilize LPA to identify combinations of resilience across three domains in adolescents.
- Co‐twin control analyses parse genetic versus environmental contributions to links between social processes (i.e., parenting, peer influences) and resilience.

**What's clinically relevant**

- Resilience to neighborhood disadvantage is heterogeneous. Thus, youth may need more personalized interventions to bolster resilience in multiple areas.
- Greater parental nurturance and less parent–child conflict are environmentally mediated pathways associated with greater adolescent resilience. Parenting interventions may be an especially powerful target for boosting resilience in the context of neighborhood disadvantage.

## Supporting information


**Appendix S1:** Description of behavioral measures.
**Appendix S2:** Analytic method details.
**Appendix S3:** Cumulative risk sensitivity analysis.
**Appendix S4:** Area deprivation index (ADI) score sensitivity analysis.
**Figure S1:** Area deprivation index scores in the MTwiNS sample.
**Figure S2:** Resilience latent profile analysis of youth with ADI scores of 30 or greater.
**Table S1:** Cumulative risk index indicators.
**Table S2:** Latent profile analysis model fit statistics.
**Table S3:** ADI score descriptive statistics.
**Table S4:** ADI score sensitivity analyses: latent profile analysis model fit statistics.
**Table S5:** ADI score sensitivity analysis: three‐profile solution analysis of variance (ANOVA) comparison of resilience indicators.
**Table S6:** ADI score sensitivity analysis: Bolck, Croon, and Hagenaars (BCH) 3‐step analysis for demographic characteristics and parenting, peer, and neighborhood social processes.
**Table S7:** Cumulative risk latent profile analysis model fit statistics.
**Table S8:** Co‐Twin control regression analyses reveal patterns of environmental effects for parenting and mixed genetic and environmental effects for peers.

## Data Availability

The raw data that support the findings of this study is shared via the NIMH Data Archive.
