# Supplementary material for: A person‐centered and genetically informed approach toward characterizing multidomain resilience to neighborhood disadvantage in youth
Source: J Child Psychol Psychiatry. 2025 Oct 24;67(6):804–15. doi: 10.1111/jcpp.70068 (PMC13170636; doi:10.1111/jcpp.70068)
Supplement: Supplementary file 1 — Appendix S1: Description of behavioral measures. Appendix S2: Analytic method details. Appendix S3: Cumulative risk sensitivity analysis. Appendix S4: Area deprivation index (ADI) score sensitivity analysis. Figure S1: Area deprivation index scores in the MTwiNS sample. Figure S2: Resilience latent profile analysis of youth with ADI scores of 30 or greater. Table S1: Cumulative risk index indicators. Table S2: Latent profile analysis model fit statistics. Table S3: ADI score descriptive statistics. Table S4: ADI score sensitivity analyses: latent profile analysis model fit statistics. Table S5: ADI score sensitivity analysis: three‐profile solution analysis of variance (ANOVA) comparison of resilience indicators. Table S6: ADI score sensitivity analysis: Bolck, Croon, and Hagenaars (BCH) 3‐step analysis for demographic characteristics and parenting, peer, and neighborhood social processes. Table S7: Cumulative risk latent profile analysis model fit statistics. Table S8: Co‐Twin control regression analyses reveal patterns of environmental effects for parenting and mixed genetic and environmental effects for peers. [file JCPP-67-804-s001.docx]

**A Person-centered and Genetically-informed Approach Toward Characterizing Multidomain Resilience to Neighborhood Disadvantage in Youth**

**Supporting Information**

**Appendix S1:** Description of Behavioral Measures.

**Appendix S2:** Analytic Method Details.

1. Latent Profile Analysis
2. Co-twin Control Analysis

**Appendix S3:** Cumulative Risk Sensitivity Analysis.

**Appendix S4:** Area Deprivation Index (ADI) Score Sensitivity Analysis.

**Figure S1:** Area Deprivation Index Scores in the MTwiNS Sample.

**Figure S2:** Resilience Latent Profile Analysis of Youth with ADI Scores of 30 or Greater.

**Table S1:** Cumulative Risk Index Indicators.

**Table S2:** Latent Profile Analysis Model Fit Statistics.

**Table S3:** ADI Score Descriptive Statistics.

**Table S4:** ADI Score Sensitivity Analyses: Latent Profile Analysis Model Fit Statistics.

| **Table S5:** ADI Score Sensitivity Analysis: Three-Profile Solution Analysis of Variance (ANOVA) Comparison | |
| --- | --- |
| of Resilience Indicators. |  |

| **Table S6:** ADI Score Sensitivity Analysis: Bolck, Croon, and Hagenaars (BCH) 3-Step Analysis for | |
| --- | --- |
| Demographic Characteristics and Parenting, Peer, and Neighborhood Social Processes | . |

**Table S7:** Cumulative Risk Latent Profile Analysis Model Fit Statistics.

**Table S8:** Co-Twin Control Regression Analyses Reveal Patterns of Environmental Effects for Parenting and Mixed Genetic and Environmental Effects for Peers.

# Appendices

## Appendix S1. Description of Behavioral Measures

### Friends Questionnaire

Youth rated their friends on a 4-point scale with the following options: 1) All my friends are like that, 2) Most of my friends are like that, 3) Just a few of my friends are like that, 4) None of my friends are like that.

### Neighborhood Measures

**Informants.** Mailing packets were sent to 10 randomly chosen addresses in each twin family’s Census tract, inviting one adult resident per household to complete a survey. When an address was no longer inhabited (i.e., the letter was undeliverable), one attempt was made to find a replacement address. All participants provided informed consent.

**Neighborhood Matters Questionnaire.** For each item on the *Informal Social Control* scale, participants selected 1 of 4 responses: (1) Do nothing; (2) Complain to or discuss with other neighbors;

(3) Talk to someone who can do something about it, for example the police, a landlord, or a parent; (4) Do something directly, for example, step in and/or talk to the person or people involved.

**Area Deprivation Index (ADI).** ADI scores measure indices of concentrated disadvantage in the neighborhood via indicators of neighbors’ education, employment, income, and poverty (e.g., home ownership rates, percentage of single-parent households, percentage of families living below the poverty line, percentage of those 16 years or older unemployed). ADI scores were calculated using the Singh method, which involves summing Singh’s 17 census indicator weights by Singh’s factor score coefficients for each indicator (Singh, 2003). The ADI uses the American Community Survey (ACS) Five Year Estimates in its construction (e.g., the 2015 ADI uses the ACS data from 2015, which is a 5-year average of data obtained from 2011 to 2015). This five-year period overlapped with the timing of data collection for the twin sample. The ADI provides a national percentile ranking at the block group level from 1 to 100. These percentiles were constructed by ranking the ADI from low-to-high for the nation and grouping the block groups into bins corresponding to each 1% range of the ADI.

## Appendix S2: Analytic Method Details

### Latent Profile Analysis

**Methods.** Simulation studies support the utility of BIC and SABIC as criteria for guiding model selection, with lower values indicating better model fit (Ferguson et al., 2020). The Adjusted LMR-LRT assesses whether a model shows significantly better model fit than a model with one less profile, such that values of *p* < .05 reflect a statistically significant improvement in model fit from the previous model. Additionally, model entropy values (on a scale from 0-1) were considered, with higher values indicating better model fit (Tein et al., 2013).

**Results.** As predicted, the log-likelihood value, BIC, and SABIC decreased with the addition of each profile across all variance-covariance specifications, indicating an improvement in model fit.

However, models with Equal variances, Equal covariances, as well as the Profile-specific variances, Profile-specific covariances models had insufficient log likelihood replication, suggesting that these models were overly complex to accommodate the data. Additionally, the Equal variances, No covariances models exhibited largely different sample sizes across profiles, limiting interpretability. As such, we proceeded with comparing the solutions from the Profile-specific variances, No covariances models.

In models with profile-specific variances and no covariances, the four-profile model exhibited the lowest log-likelihood, BIC, and SABIC values. However, the Adjusted Lo-Mendell-Rubin LRT *p*-value for the four-profile solution was not significant. Additionally, the four-profile solution included a profile containing only 48 individuals, restricting opportunities for analysis of associated environmental characteristics due to limited sample size. Lastly, the four-profile solution evidenced lower entropy than the three-profile model, indicating that the classification of individuals was less stable. Thus, statistical model fit and theoretical considerations suggested that the three-profile solution was the best fitting model (see Figure 2).

### Co-Twin Control Analyses

To generate more interpretable unstandardized beta coefficients, we standardized all variables prior to co-twin control analyses. Resilience profiles were standardized from -1 to 1, with profile one

(Low Multidomain Resilience) recoded as negative one, profile two (Low Psychological, High Social

Resilience) recoded as zero, and profile three (High Multidomain Resilience) recoded as positive one.

Parenting and peer variables were z-scored to have a mean of zero and a standard deviation of one. In order to assess within-twin pair variation, we divided the mean absolute difference score within twins for the four environmental exposure variables by the phenotypic (i.e., full sample) standard deviation. The within-twin pair variation for the four variables assessed within the co-twin control models were as follows: Friend Popularity: 87.79%; Friend Drug-Related Behaviors: 48.79%; Parent-child Conflict: 60.52%; and Parental Involvement: 80.20%. Otherwise stated, in co-twin control models in which parenting was significantly linked to resilience outcomes, twin pairs showed 60-80% of the variation in parenting experiences compared to unrelated individuals. For friend experiences, twin pairs showed 49-88% of the variation in friend characteristics compared to the full sample. As expected, twins differed less on environmental exposures than unrelated individuals, but twin pairs still demonstrated a meaningful proportion of variability.

**Appendix S3: Cumulative Risk Sensitivity Analysis.**

### Questionnaires

The cumulative risk index was composed of eight indicators collected from youth and their parents (Bezek et al., 2024). Youth completed the KID-Screen for Adolescent Violence Exposure to assess for three areas of community violence exposure: traumatic violence, indirect violence, and physical/verbal abuse (Flowers et al., 2000). Participants met criteria for exposure if they endorsed any items on the traumatic violence OR physical/verbal abuse subscales, OR if their score on the indirect violence subscale fell one standard deviation above the mean. Youth completed the Child Trauma Questionnaire to screen for five areas of trauma exposure: sexual abuse, physical abuse, emotional abuse, emotional neglect, and physical neglect (Bernstein et al., 2003). Youth met criteria for exposure if they scored above a pre-determined cutoff score indicating moderate to extreme levels of exposure on ANY of the five subscales (Zhang et al., 2020). Youth completed the Parental Environment Questionnaire to screen for exposure to harsh parenting (i.e., corporal punishment). Youth met criteria for exposure to harsh parenting if they endorsed the item “My parents sometimes hit me in anger” on the Parental Environment Questionnaire (Elkins et al., 1997). Parents completed the Conflict Tactics Scale (CTS) to assess for inter-partner violence. Youth met criteria for exposure to inter-parental aggression if either the primary (typically mother) or alternate (typically father) caregiver’s combined sum on the violence/physical aggression and verbal aggression subscales of the Conflict Tactics Scale (Straus, 1979) fell one standard deviation above the mean. Youth completed the Adult Child Relationship Scale to assess for exposure to parent-child conflict. Youth met criteria for exposure if they scored one standard deviation above the mean on the conflict subscale (Pianta & Nimetz, 1991). Youth met criteria for exposure to stressful life events if their self-reported total score on the Adolescent Life Events Scale fell at least one standard deviation above the mean (Aggarwal et al., 2007). To measure stressful events specifically, we calculated youth’s total scores using only the items from the Adolescent Life Events Scale describing explicitly negative life events (N = 36 items). Youth met criteria for exposure to parental depression if their parent’s self-reported score on the Beck Depression Inventory fell above a score of 13, which captures mild, moderate, and severe depression (Beck et al., 1961). Given that the Beck

Depression Inventory assesses for depression symptoms in the past two weeks, this measure of exposure to parental depression is acute rather than chronic. Finally, youth met criteria for income-related adversity if their parent reported an annual household income at or below 200% of the federal poverty line. This cut-off point encompasses families living at or near poverty, who are federally classified as low-income and eligible for federally-based benefits (*Population Reference Bureau*, 2022). Parent-reported depression and family income included in all final analyses were collected from youth’s primary caregiver.

### Cumulative Risk Sensitivity Analysis

Though the entire sample faced neighborhood adversity as determined via sampling criteria, there is likely substantial heterogeneity within the sample in terms of how much cumulative adversity youth experience (i.e., specific exposures). As much of the resilience literature has focused on specific or cumulative exposures to adversity, we also examined whether resilience profiles might differ when only focusing on youth with higher exposure to specific adversities. We ran latent profile analyses with profiles-specific variances, and no covariances for one, two, three, four, and five-profile solutions in a subsample of 564 youth with higher exposure to adversity (i.e., a score of one or more on the cumulative risk index). We found that the best fitting model included the same three profiles extracted in the full sample (see Table S7), indicating that resilience profiles appear to cluster similarly across the whole sample, as in the high adversity exposure subsample. Given similar results across both sampling approaches, analyses utilized the entire MTwiNS sample (n = 704) to maintain our focus on neighborhood disadvantage and provide greater statistical power.

**Appendix S4: Area Deprivation Index (ADI) Score Sensitivity Analysis.**

### Latent Profile Analysis

Neighborhoods with Area Deprivation Index (ADI) scores of 30 or greater experience more substantial disadvantage based on 17 indicators of poverty housing, employment, and education (Kind et al., 2014; Suarez et al., 2025). In order to test resilience profile robustness at higher levels of disadvantage exposure, an additional set of latent profile analyses were conducted in a sample of 636 adolescents with ADI scores of 30 or greater. In line with original analyses, a three-profile model fit the data best compared to two- and four-profile solutions (Table S4). Specifically, the Bayesian Information Criterion (BIC) and SABIC criteria were lower in the three-profile solution compared to the two-profile solution, indicating better model fit. Additionally, the log likelihood value did not replicate within the four-profile solution and the Adjusted Lo Mendell Rubin Likelihood Ratio Test was unable to be computed, suggesting that the four-profile solution was overly complex for the data even after increasing the number of model starts.

As depicted in Table S5, follow-up ANOVAs comparing profiles on the 10 resilience indicators demonstrated a nearly identical pattern of profile differences to the original sample. The youth-reported social activities indicator showed mildly attenuated differences; however, all profiles were shown to differ on social activities at a *p* < .05 level in Bonferroni post-hoc comparisons. Only three individuals from the original latent profile analysis shifted profiles within the sample of 636 youth, demonstrating nearly identical profile replication. One participant moved from the Low Multidomain profile to the Low Psychological, High Social Resilience profile and two participants moved from the Low Multidomain profile to the High Multidomain Resilience profile.

### BCH-3 Step Analyses

BCH 3-Step models comparing latent profiles on parenting, peer, and neighborhood social processes showed largely consistent results for the elevated ADI subsample (Table S6). Profile differences in parent-child conflict, friend popularity, neighborhood norms, and neighborhood social cohesion remained consistent, and profile differences in parental involvement became stronger. Differences between profiles on neighborhood informal social control and peer drug-taking behaviors were mildly attenuated; however, the direction of the associations remained consistent. Altogether, the pattern of results remains largely consistent, with only minor reductions in effect size that likely reflect reduced power rather than a substantive change in the relationship between environmental exposures and resilience.

**References**

Aggarwal, S., Prabhu, C. H., Anand, L. C. A., & Kotwal, L. C. A. (2007). Stressful life events among adolescents: The development of a new measure. *Indian journal of psychiatry*, *49*(2), 96.

Beck, A. T., Ward, C. H., Mendelson, M., Mock, J., & Erbaugh, J. (1961). An inventory for measuring depression. *Archives of general psychiatry*, *4*(6), 561-571.

Bernstein, D. P., Stein, J. A., Newcomb, M. D., Walker, E., Pogge, D., Ahluvalia, T., ... & Zule, W. (2003).

Development and validation of a brief screening version of the Childhood Trauma Questionnaire.

*Child abuse & neglect, 27*(2), 169-190.

Elkins, I. J., McGue, M., & Iacono, W. G. (1997). Genetic and environmental influences on parent-son relationships: Evidence for increasing genetic influence during adolescence. *Developmental*

*Psychology, 33,* 351-363.

Flowers, A. L., Hastings, T. L., & Kelley, M. L. (2000). Development of a screening instrument for exposure to violence in children: The KID-SAVE. *Journal of Psychopathology and Behavioral Assessment, 22*(1), 91-104.

Pianta, R. C., & Nimetz, S. L. (1991). Relationships between children and teachers: Associations with classroom and home behavior. *Journal of Applied Developmental Psychology*, 12, 379–393.

Population reference bureau (2022) https://www.prb.org/resources/how-poverty-in-the-united-states- is-measured-and-why-it-matters/

Singh, G. K. (2003). Area deprivation and widening inequalities in US mortality, 1969–1998. *American journal of public health*, *93*(7), 1137-1143.

Straus Murray, A. (1979). Measuring intrafamily conflict and violence: The conflict tactics (CT) scales.

*Journal of Marriage and the Family, 41*(1), 75-88.

Suarez, G. L., Burt, S. A., Gard, A. M., Burton, J., Clark, D. A., Klump, K. L., & Hyde, L. W. (2022). The impact of neighborhood disadvantage on amygdala reactivity: Pathways through neighborhood social processes. *Developmental Cognitive Neuroscience*, *54*, 101061.

Suarez, G. L., Bezek, J. L., Westerman, H. B., Hanson, J. L., Klump, K. L., Burt, S. A., & Hyde, L. W. (2025). Structural Brain Correlates of Multi-Domain Resilience Among Youth Exposed to Neighborhood

Disadvantage. *Biological Psychiatry Global Open Science*, 100550.

Zhang, S., Lin, X., Liu, J., Pan, Y., Zeng, X., Chen, F., & Wu, J. (2020). Prevalence of childhood trauma measured by the short form of the Childhood Trauma Questionnaire in people with substance use disorder: A meta-analysis. *Psychiatry research*, *294*, 113524.

# Supporting Figures

**Figure S1**

***Area Deprivation Index (ADI) Scores in the MTwiNS Sample***


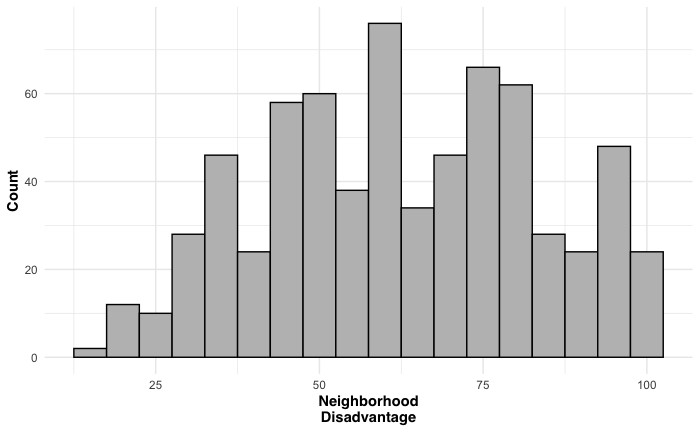


*Note.* N = 708. Area Deprivation Index (ADI) scores measure concentrated disadvantage in the neighborhood via 17 indicators of neighbors’ education, employment, income, and poverty (e.g., home ownership rates, percentage of single-parent households, percentage of families living below the poverty line, percentage of those 16 years or older unemployed). ADI scores are derived from the American Community Survey Five-Year Estimates and provide a national percentile ranking at the block group level from 1 to 100 with group 1 reflecting the lowest level of disadvantage in the nation, whereas group 100 represents the highest level of disadvantage. In the MTwiNS sample, 90% of youth live in neighborhoods with ADI scores of 30 or greater.

**Figure S2**

***Resilience Latent Profile Analysis of Youth with ADI Scores of 30 or Greater***


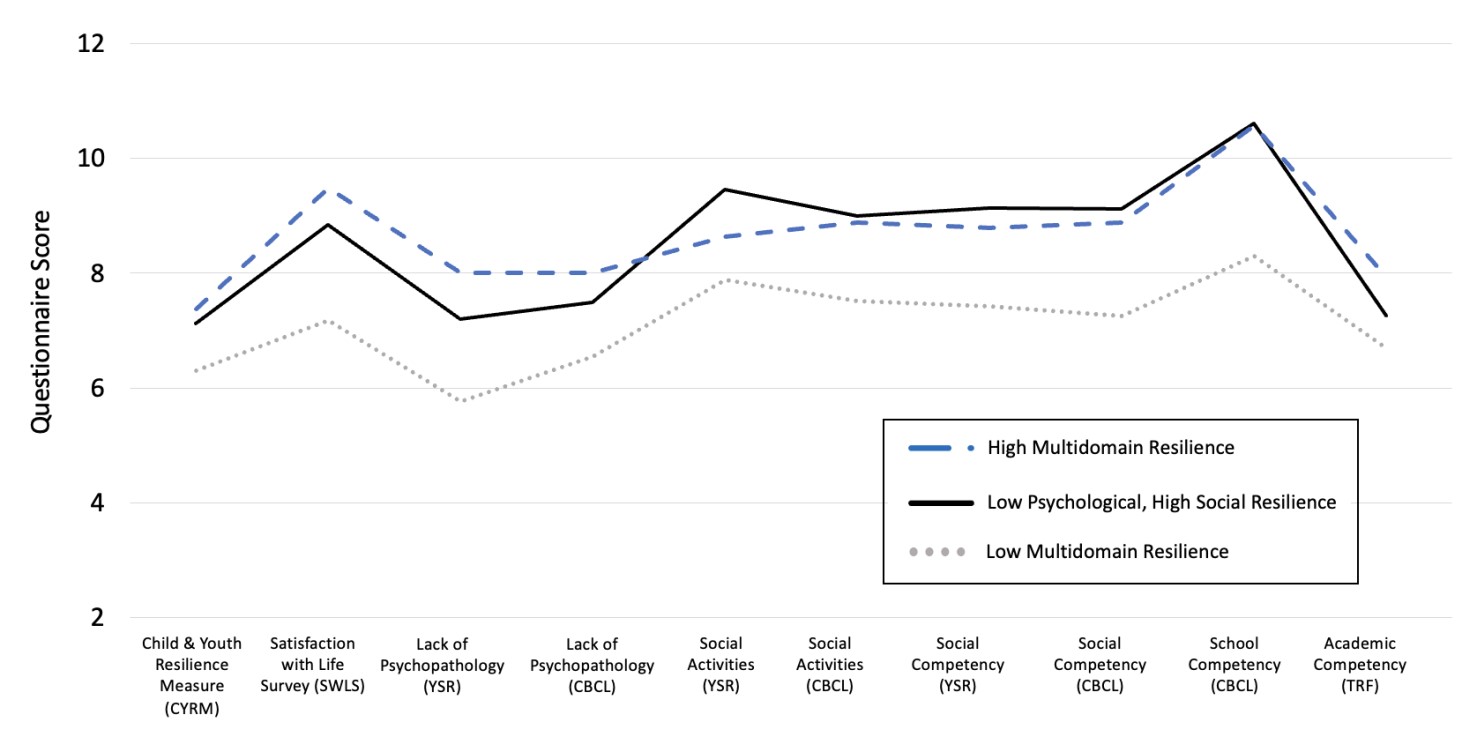


*Note.* Evaluation of model fit statistics across one, two, three, and four-profile solutions revealed that the three-profile model with profile-specific variance and no covariances fit the data best (N = 636). The three resilience profiles were labeled as: 1) Low Multidomain Resilience, 2) Low Psychological, High Social Resilience, and 3) High Multidomain Resilience. The Low Multidomain Resilience Profile reported poorer functioning on all ten indicators of resilience across psychological, social, and academic domains. The Low Psychological, High Social Resilience exhibited lower levels of psychological resilience as well as lower academic competency compared to youth in the High Multidomain resilience group. However, these youth reported the highest activity engagement and equally high social resilience to peers in profile three. The High Multidomain Resilience group showed the highest levels of psychological resilience compared to both other profiles, as well as the highest academic competency as rated by teachers. Child-reported forms include the CYRM, SWLS, and YSR. Parent-reported forms include the CBCL. Teacher-reported forms include the TRF. YSR = Youth Self-report Form, CBCL = Child Behavior Checklist, TRF = Teacher Report Form. Y axis represents individuals’ score on each questionnaire. In order to more readily compare questionnaire scores across profiles, we rescaled four variables in order to create a similar range for all measures included in the latent profile analysis. Specifically, we divided youth’s total scores for the Child & Youth Resilience Measure by 10, divided youth’s scores on the Satisfaction with Life Scale by three, multiplied parent’s scores on the School Competency measure by two and multiplied teacher’s scores on the Academic Competency measure by two.

# Supporting Tables

## Table S1

### Cumulative Risk Index Indicators

| **Risk Indicator** | **Measure** | **Subscales** | **Exposure Cut-Off Score** | **N** | **Mean (SD)** | **Participants**  **Endorsing**  **Risk Exposure** | **Citation** |
| --- | --- | --- | --- | --- | --- | --- | --- |
| Exposure to  Community  Violence | KID-Screen for  Adolescent  Violence Exposure | Traumatic Violence  Indirect Community  Violence  Physical/Verbal Abuse  Outside of the Home | Any items endorsed  ≥ 1 SD above Mean    Any items endorsed | 675  679    683 | 0.11 (0.61)  2.86 (3.68)    0.52 (0.90) | 244 | Flowers et al., 2000 |
| Abuse/Neglect | Child Trauma  Questionnaire  (CTQ) | Sexual Abuse  Physical Abuse  Emotional Abuse  Emotional Neglect | ≥ 8  ≥ 10  ≥ 13  ≥ 15 | 701  705  707  707 | 5.31 (1.65) 3.17 (0.76) 7.53 (2.93)  8.68 (3.72) | 149 | Bernstein et al., 2003 |
|  |  | Physical Neglect | ≥ 10 | 706 | 8.68 (3.72) |  |  |
| Harsh  Parenting | Parental  Environment  Questionnaire  (PEQ) | Corporal Punishment Item | Item rated as definitely or probably true | 626 | 3.84 (0.50) | 19 | Elkins et al.,  1997 |
| Inter-partner Violence | Conflict Tactics Scale (CTS) | Violence/Physical &  Verbal Aggression | Either parent reported ≥ 1 SD above Mean | 684 (Primary caregiver)  514 (Alternate caregiver) | 5.05 (5.52)    4.36 (4.53) | 138 | Straus, 1979 |
| Parent-Child Conflict | Adult Child  Relationship Scale  (ACRS) | Relationship Conflict | ≥ 1 SD above Mean | 677 | 20.85 (7.12) | 118 | Pianta & Nimetz, 1991 |
| Stressful Life Events | Adolescent Life  Events Scale (ALES) | Negative Life Events | ≥ 1 SD above Mean | 692 | 5.94 (4.29) | 100 | Aggarwal et al., 2007 |
| Parental  Depression | Beck Depression Inventory (BDI) | Depression Total Score | ≥ 13 | 708 | 6.37 (6.41) | 80 | Beck et al., 1961 |
| Family Income | Parent  Demographic  Questionnaire | Annual Family Income |  200% of poverty line | 694 | $60,000 $69,984 | 333 | Suarez et al., 2022 |

*Note.* Final cumulative risk scores ranged from 0 to 7 (M = 1.72, SD = 1.38). 698 subjects had data for at least six out of eight risk indicators and thus received a final cumulative risk score.

## Table S2

### Latent Profile Analysis Model Fit Statistics

| **Model** | **Number of**  **Profiles** | **Number of Parameters** | **Log Likelihood** | **Bayesian Information Criteria (BIC)** | **Sample Adjusted BIC** | **Entropy** | **Adjusted Lo Mendell Rubin** |
| --- | --- | --- | --- | --- | --- | --- | --- |
| Equal Variances, No Covariances | 1  2  3  4 | 20  31  42  53 | -9439.981  -9052.970  -8824.168  -8660.799 | 19011.097  18309.200  17923.720  17669.107 | 18947.592  18210.768  17790.361  17500.820 | ----  0.982 0.779  0.820 | -----  0.0995 0.0252  0.2495 |
|  | 5 | 64 | -8509.054 | 17437.741 | 17234.527 | 0.851 | 0.4308 |
|  | 6 | 75 | N/A | 17320.470 | 17082.329 | 0.867 | 0.6137 |
| Equal Variances, Equal Covariances | 1  2  3  4 | 65  76  87  98 | -8820.023  -8526.219  -8366.572  -8192.302 | 18066.237  17550.7540  17303.5840  17027.1680 | 17859.848  17309.437  17027.341  16715.997 | ----  0.994 0.982  0.994 | -----  0.0211 0.5650  0.7478 |
|  | 5 | 109 | -8083.473 | 16881.6360 | 16535.537 | 0.988 | 0.5210 |
| Profile-Specific Variances,  Profile-Specific Covariances | 1  2  3  4 | 65  131  196  262 | -8820.023 N/A  N/A  N/A | 18066.237  17150.0940  14392.4380  14674.4230 | 17859.848  16734.140  13770.096  13842.516 | ----  0.941 0.958  0.942 | -----  0.0398 0.2398  0.2398 |

*Note.* Results for latent profile analyses conducted with the full 704 twin sample. Values marked as N/A indicate log likelihood values that did not replicate.

**Table S3**

*ADI Score Descriptive Statistics*

| **Sample** | **Profile** | **N** | **Mean ADI Score** | **SD** | **Minimum** | **Maximum** | **Skew** | **Kurtosis** |
| --- | --- | --- | --- | --- | --- | --- | --- | --- |
| Full Sample (*N* = 708) | 1  2 | 127  136 | 64.60  60.84 | 19.66  20.12 | 22  21 | 99  98 | -0.09  0.18 | -0.83  -0.89 |
|  | 3 | 442 | 59.06 | 21.51 | 12 | 99 | 0.04 | -0.95 |
| Higher ADI  Subsample  (*n* = 636) | 1  2 | 113  129 | 65.94  62.64 | 18.55  18.82 | 31  31 | 99  98 | 0.00  0.31 | -0.91  -0.98 |
|  | 3 | 394 | 62.65 | 19.21 | 30 | 99 | 0.17 | -1.06 |

*Note.* Profile 1 = Low Multidomain Resilience, Profile 2 = Low Psychological Resilience, High Social Resilience, Profile 3 = High Multidomain Resilience.

**Table S4**

*ADI Score Sensitivity Analysis: Latent Profile Analysis Model Fit Statistics*

| **Number of**  **Profiles** | **Number of Parameters** | **Log Likelihood** | **Bayesian Information Criterion (BIC)** | **Sample-adjusted BIC** | **Entropy** | **Adjusted Lo Mendell Rubin Likelihood Ratio Test** |
| --- | --- | --- | --- | --- | --- | --- |
| 1-profile | 20 | -8158.492 | 16446.088 | 16382.590 | ----- | ------ |
| 2-profile | 39 | -5425.168 | 11102.088 | 10978.267 | 0.989 | 0.0000 |
| **3-profile** | **60** | **-5257.972** | **10903.256** | **10712.761** | **0.926** | **0.0100** |
| 4-profile | 79 | -5858.630* | 12227.221 | 11976.403 | 0.971 | Not computed^ |
| 5-profile | 100 | -5824.960* | 12295.440 | 11977.949 | 0.956 | 0.0169 |

*Note.* Latent profile analyses were conducted with a minimum ADI score of 30 (N = 636). All models were run with profile-specific variance and no covariances. *Log Likelihood values were not replicated for the 4-profile and 5-profile solutions despite increasing the number of random starts to 1500. ^The Adjusted LRT value could not be computed for the four-profile solution, further reflecting that the model is overly complex for the data and the four-profile solution does not fit the data better than the three-profile solution.

**Table S5**

*ADI Score Sensitivity Analysis: Three-Profile Solution Analysis of Variance (ANOVA) Comparison of Resilience Indicators*

| **Profile 1:**  Low  Multidomain  Resilience  (n = 127) | **Profile 2:**  Low Psych,  High Social  Resilience  (n = 135) | **Profile 3:**  High  Multidomain  Resilience  (n = 442) | ***df*** | **F** | **2** | **Tukey**  **Post-hoc**  **Differences** |
| --- | --- | --- | --- | --- | --- | --- |

|  | **M** | **SD** | **M** | **SD** | **M** | **SD** |  |  |  |  |
| --- | --- | --- | --- | --- | --- | --- | --- | --- | --- | --- |
| Child & Youth Resilience  Measure | 6.301 | 1.083 | 7.120 | 0.805 | 7.378 | 0.836 | 624 | 118.6*** | .16 | 1  2  3 |
| Satisfaction with Life Survey | 7.179 | 2.318 | 8.836 | 1.579 | 9.474 | 1.656 | 407 | 93.68*** | .19 | 1  2  3 |
| Lack of Psychopathology (YSR) | 5.765 | 2.022 | 7.205 | 0.641 | 8.000 | 0.032 | 625 | 559.9*** | .47 | 1  2  3 |
| Lack of Psychopathology (CBCL) | 6.546 | 1.713 | 7.488 | 0.548 | 8.000 | 0.032 | 627 | 333.4*** | .35 | 1  2  3 |
| Social Activities (YSR) | 7.876 | 2.530 | 9.459 | 2.113 | 8.637 | 2.472 | 418 | 3.269^+^ | .01 | 1  2  3 |
| Social Activities (CBCL) | 7.509 | 2.892 | 9.001 | 2.396 | 8.877 | 2.223 | 416 | 17.64*** | .04 | 1  2, 3 |
| Social Competency (YSR) | 7.423 | 2.539 | 9.137 | 2.228 | 8.793 | 2.495 | 418 | 13.56*** | .03 | 1  2, 3 |
| Social Competency (CBCL) | 7.254 | 2.967 | 9.114 | 2.311 | 8.881 | 2.407 | 415 | 20.87*** | .05 | 1  2, 3 |
| School Competency (CBCL) | 8.301 | 2.693 | 10.602 | 1.315 | 10.567 | 1.653 | 416 | 84.08*** | .17 | 1  2, 3 |
| Academic Performance (TFR) | 6.697 | 1.960 | 7.253 | 1.932 | 7.938 | 1.933 | 309 | 19.59*** | .06 | 1, 2  3 |

*Note.* M = mean, SD = standard deviation, YSR = Youth Self-report Form, CBCL = Child Behavior Checklist, TRF = Teacher Report Form. ^+^*p* = .07, ****p* < .001.

**Table S6**

ADI Score Sensitivity Analysis: Bolck, Croon, and Hagenaars (BCH) 3-Step Analysis for Demographic Characteristics and Parenting, Peer, and Neighborhood Social Processes

|  | **Low Multidomain Resilience** *(Profile 1)* **vs. Low Psych Resilience, High Social**  **Resilience** *(Profile 2)*      Estimate | **Low Multidomain Resilience**  *(Profile 1)* **vs. High Multidomain**  **Resilience** *(Profile 3)*      Estimate | **Low Psych Resilience, High**  **Social Resilience** *(Profile 2)* **vs.**  **High Multidomain Resilience**  *(Profile 3)*    Estimate |
| --- | --- | --- | --- |
| *Demographic Characteristics*  Age | **0.971**** | **0.615*** | -0.356 |
| Gender | 0.114 | 0.099 | -0.015 |
| Race | 0.036 | 0.062 | 0.025 |
| Family Income | **-0.984*** | **-0.961*** | 0.023 |
| National Area Deprivation Index Score | 3.888 | 3.446 | -0.442 |
| *Environmental Variables*  Neighborhood Social Cohesion | 23.468**^†^** | 4.678 | **-18.790*** |
| Neighborhood Norms | **17.544*** | 4.178 | **-13.366*** |
| Neighborhood Informal Social Control | 7.096 | 4.261 | -2.834 |
| Parental Involvement (youth-report) | **-33.464***** | **-16.152*** | **17.312**** |
| Parental Involvement (parent-report) | -4.772 | -1.750 | 3.022 |
| Parental Conflict (youth-report) | 18.939 | 14.189 | -4.749 |
| Parental Conflict (parent-report) | -3.078 | 8.531 | **11.609*** |
| Friend Popularity | -2.808 | **-4.216*** | -1.408 |
| Friend Academics | -6.292**^†^** | -4.260 | 2.032 |
| Friend Delinquency | 3.490 | 2.178 | -1.312 |
| Friend Drug-taking Behaviors | -3.767**^†^** | **-3.611*** | 0.156 |

*Note.* BCH 3-step models comparing mean levels of demographic characteristics and environmental variables across the three latent profiles with a sample in which the minimum ADI score is 30 (N = 636). For each column with the format A profile vs. B profile, negative values indicate that the A profile scored lower than the B profile. Positive values indicate that the A profile scored higher than the B profile. Note that the Low Multidomain Resilience profile shows lower scores on the friend popularity and drug-taking behaviors subscales because the scale is scored such that lower scores indicate *more* of the youth’s friends demonstrate the behavior. * = *p*<.05, ** = *p*<.01, *** = *p*<.001, **^†^** = .05 < *p* <.07

## Table S7

### Cumulative Risk Latent Profile Analysis Model Fit Statistics

| **Number of**  **Profiles** | **Number of Parameters** | **Log Likelihood** | **Bayesian Information Criterion (BIC)** | **Sample-adjusted BIC** | **Entropy** | **Adjusted Lo Mendell**  **Rubin Likelihood**  **Ratio Test** |
| --- | --- | --- | --- | --- | --- | --- |
| 1-profile | 20 | -7320.932 | 14768.566 | 14705.075 | ---- | ----- |
| 2-profile | 39 | -5015.272 | 10277.612 | 10153.806 | 0.990 | 0.0000 |
| **3-profile** | **60** | **-4877.921** | **10135.946** | **9945.475** | **0.918** | **0.0204** |
| 4-profile | 81 | -4808.584 | 10130.308 | 9873.172 | 0.897 | 0.5307 |
| 5-profile | 101 | Not Replicated | 11611.730 | 11219.104 | 0.919 | 0.2398 |

*Note.* Latent profile analyses were conducted with youth who reported a score of one or more on the cumulative risk index, reflecting additional exposures to community and family-level risk. All models were run with profile-specific variance and no covariance. N = 564.

## Table S8

### Co-Twin Control Regression Analyses Reveal Patterns of Environmental Effects for Parenting and Mixed Genetic and Environmental Effects for Peers

|  | **Individual Level** | |  | **Within Dizygotic Pairs** | |  | **Within Monozygotic Pairs** | |  |
| --- | --- | --- | --- | --- | --- | --- | --- | --- | --- |
|  | Estimate | *p*-value | N | Estimate | *p*-value | N | Estimate | *p*-value | *N* |
| Parental Conflict *(Parent Report)* | -0.219 | <.001 | 627 | -0.155 | 0.017 | 387 | -0.192 | 0.069 | 240 |
| Parental  Involvement  *(Youth Report)* | 0.274 | <.001 | 622 | 0.164 | 0.003 | 385 | 0.164 | 0.05 | 237 |
| Friend Drug-related Behavior *(Youth Report)* | -0.119 | <.001 | 658 | 0.009 | 0.875 | 405 | 0.017 | 0.845 | 253 |
| Friend Popularity *(Youth Report)* | 0.093 | 0.001 | 663 | 0.074 | 0.106 | 409 | 0.014 | 0.847 | 254 |

*Note.* Co-twin control models improve the causal resolution of analyses by splitting regression effects separately by zygosity both within and between twin pairs. Within monozygotic twins, we are able to observe genetically identical individuals with differing levels of exposure to the variable of interest (i.e., parenting, friend characteristics). The interpretive framework depicted in Figure 1 reveals how the magnitude of the effect of X on Y (e.g., parent-child conflict on resilience) differs across the entire sample, within monozygotic twins, and within dizygotic twins. Analyses involve running three multi-level models per environmental exposure. First, we calculate the within-pair effect estimate for monozygotic twins (i.e., we are regressing the within-pair difference in outcomes on the difference in exposures). Second, we calculate the within-pair effect for dizygotic twins. Lastly, we calculate the individual level effect, which represents the effect between twin pairs. All models include only subjects with complete data, as the co-twin control models did not impute missing data. Sample size for each model is reported in the “N” column.
